# Rare forms of genetic paediatric adrenal insufficiency: Excluding congenital adrenal hyperplasia

**DOI:** 10.1007/s11154-023-09784-7

**Published:** 2023-02-10

**Authors:** Valeria Hasenmajer, Rosario Ferrigno, Marianna Minnetti, Bianca Pellegrini, Andrea M. Isidori, Andrea Lenzi, Mariacarolina Salerno, Marco Cappa, Li Chan, Maria Cristina De Martino, Martin O. Savage

**Affiliations:** 1 Department of Experimental Medicine, Sapienza University of Rome, Rome, Italy; 2 UOSD Auxology and Endocrinology, Department of Pediatric, AORN Santobono-Pausilipon, Naples, Italy; 3 Dipartimento Di Medicina Clinica E Chirurgia, Federico II University, Naples, Italy; 4 Endocrinology Unit, Pediatric University Department, Bambino Gesù Children’s Hospital, Rome, Italy; 5 Endocrinology Centre, William Harvey Research Institute, Barts and the London School of Medicine and Dentistry, Queen Mary University of London, Charterhouse Square, London, EC1M 6BQ UK

**Keywords:** Adrenal insufficiency, Cortisol, Aldosteron, Intrauterine growth retardation syndromes, Addison, Adrenoleukodystrophy, Allgrove syndrome, Triple A syndrome, Familial glucocorticoid deficiency, Pseudohypoaldosteronism, Steroidogenesis, Aldosterone Synthase

## Abstract

Adrenal insufficiency (AI) is a severe endocrine disorder characterized by insufficient glucocorticoid (GC) and/or mineralocorticoid (MC) secretion by the adrenal glands, due to impaired adrenal function (primary adrenal insufficiency, PAI) or to insufficient adrenal stimulation by pituitary ACTH (secondary adrenal insufficiency, SAI) or tertiary adrenal insufficiency due to hypothalamic dysfunction. In this review, we describe rare genetic causes of PAI with isolated GC or combined GC and MC deficiencies and we also describe rare syndromes of isolated MC deficiency. In children, the most frequent cause of PAI is congenital adrenal hyperplasia (CAH), a group of adrenal disorders related to steroidogenic enzyme deficiencies, which will not be included in this review. Less frequently, several rare diseases can cause PAI, either affecting exclusively the adrenal glands or with systemic involvement. The diagnosis of these diseases is often challenging, due to the heterogeneity of their clinical presentation and to their rarity. Therefore, the current review aims to provide an overview on these rare genetic forms of paediatric PAI, offering a review of genetic and clinical features and a summary of diagnostic and therapeutic approaches, promoting awareness among practitioners, and favoring early diagnosis and optimal clinical management in suspect cases.

## Introduction

Adrenal insufficiency (AI) is a severe endocrine disorder characterized by insufficient glucocorticoid (GC) and/or mineralocorticoid (MC) secretion by the adrenal glands, due to impaired adrenal function (primary adrenal insufficiency, PAI) or to insufficient adrenal stimulation by pituitary ACTH (secondary adrenal insufficiency, SAI), or hypothalamic dysfunction (tertiary adrenal insufficiency) [1]. In children, the most frequent cause of PAI is congenital adrenal hyperplasia (CAH), a group of adrenal disorders related to steroidogenic enzyme deficiencies [2]. The most common cause of CAH is 21OH-hydroxylase deficiency (21OH-CAH), with an incidence of 1:14,000–1:18,000 newborns, characterized by adrenal androgen excess due to the accumulation of 17OH-progesterone and other adrenal precursors with androgenic properties [2]. However, CAH is not the only cause of PAI in children. Many rare genetic diseases, either affecting exclusively the adrenal glands or with systemic involvement, have now been recognized as PAI causes. Their diagnosis is often challenging, due to the heterogeneity of the clinical presentation and to their low incidence and prevalence among the general population. Therefore, the current review aims to provide an overview of rare forms of paediatric PAI, offering a discussion of genetic and clinical features and a summary of diagnostic and therapeutic approaches, in order to promote awareness among practitioners and to favour early diagnosis and optimal treatment in suspect cases. A list of non-genetic causes of primary and secondary adrenal insufficiency is shown in Table 1. The genetic causes of PAI will be described in Table 2 and a list of causes of secondary adrenal insufficiency are shown in Table 3.

**Table 1:** Non-genetic causes of adrenal insufficiency

Primary adrenal insufficiency	
Infections leading to adrenalitis	Tuberculosis
	HIV, CMV
	Cryptococcosis, histoplasmosis, coccidiomycosis, candidiasis, african trypanomiasis
	Treponema pallidum
Bilateral adrenal haemorrhage	Septic shock, specifically meningococcal sepsis (Waterhouse-Friderichsen syndrome)
	Anticoagulants
	Trauma
Adrenal infarction	Primary antiphospholipid syndrome
	Anti-cardiolipin syndrome
	Lupus anti-coagulant syndrome
Adrenal infiltration	Autoimmune infiltration
	Adrenal metastases
	Primary adrenal lymphoma
	Sarcoidosis
	Amyloidosis
	Hemochromatosis
Bilateral adrenalectomy	Management of intractable Cushing's disease or ectopic ACTH secretion
	Following nephrectomy for tumour
	Treatment for bilateral pheochromocytoma
Drug-induced AI	Mitotane (steroid synthesis inhibitor/adrenolytic agent)
	Mifepristone (GC antagonist)
	Abiraterone acetate (steroid synthesis inhibitor)
	Trilostane (steroid synthesis inhibitor)
	Etomidate (inhibit cortisol synthesis)
	Ketaconazole/fluconazole (inhibit cortisol synthesis)
	Suramin (steroid synthesis inhibitor/adrenolytic agent)
	Aminoglutethiamide (inhibit cortisol synthesis)
	Phenytoin (increased cortisol metabolism)
	Phenobarbital (increased cortisol metabolism)
	Rifampicin (enhanced autoimmunity)
	CTLA-4 inhibitors (enhanced autoimmunity)
Secondary adrenal insufficiency	
Iatrogenic	Adrenal suppression following exogenous steroid treatment
	Adrenal suppression folllowing endogenous hypersecretion due to Cushing's syndrome
	Following TSS surgery for ACTH producing adenoma
	Pituitary irradiation and radiotherapy for brain tumours and craniospinal irradiation for other tumours
Tumours	Pituitary tumours (mostly adenomas, rarely carcinomas)
	Tumours affecting the hypothalamic-pituitary region (Craniopharyngioma, meningioma, ependymoma, intra/suprasellar metastasis
Infiltration/deposition	Tuberculosis, actinomycosis, sarcoidosis, histiocytosis X, Wegener’s granulomatosis, Hemochromatosis
Autoimmune	Autoimmune hypophysitis; most frequently in relation to pregnancy; commonly associated with panhypopituitarism, but also presenting with isolated ACTH deficiency only
Infarction/haemorrahage	Pituitary apoplexy/necrosis
	Postpartum pituitary infarction (Sheehan's syndrome)
Trauma	Pituitary stalk lesions
	Traumatic brain injury

**Table 2:** Genetic causes of primary adrenal insufficiency

Disorder	Gene	OMIM	Mechanism of action	InheritancAedditional phenotypic features
Defects in ACTH signalling				
Familial glucocorticoid deficiency 1	MC2R	202200 Loss of function	AR	Isolated GC deficiency occasionally mild MC deficiency, absent adrenarche and tall stature
Familial glucocorticoid deficiency 2	MRAP	609196 Loss of function	AR	Isolated GC deficiency occasionally mild derangement of RAA
Defects affecting the steroidogenic pathways				
Congenital lipoid adrenal hyperplasia	StAR	600617 Loss of function	AR	46,XY sex reversal, partial or complete, gonadal insufficiency
Congenital lipoid adrenal hyperplasia due to P450 SCC deficiency	CYP11A1	118485 Loss of function	AR	46,XY sex reversal, partial or complete, gonadal insufficiency
Congenital adrenal hyperplasia due to 3beta-HSD deficiency	HSD3B2	613890 Loss of function	AR	46XY Disorders of sexual development, 46XX Disorders of sexual development,
Congenital adrenal hyperplasia due to 21-hydroxylase deficiency	CYP21A2	613815 Loss of function	AR	46XX Disorders of sexual development, androgen excess
Congenital adrenal hyperplasia due to 11beta hydroxylase deficiency	CYP11B1	610613 Loss of function	AR	46XX Disorders of sexual development, androgen excess, hypertension
Congential adrenal hyperplasia due to 17α-hydroxylase deficiency	CYP17A1	609300 Loss of function	AR	46XY DSD, gonadal insufficiency, hypertension
Congenital adrenal hyperplasia due to P450 oxidoreductase deficiency	POR	124015 Loss of function	AR	46,XY DSD, 46,XX DSD, gonadal insufficiency, Antley–Bixler skeletal malformation, skeletal system, drug metabolism, developmental delay
Alterations in antioxidant mechanisms				
Familial glucocorticoid deficiency 4, with or without mineralocorticoid deficiency	NNT	607878 Loss of function	AR	Variable presentation with or without mineralocorticoid deficiency
Familial Glucocorticoid deficiency	GPX1	138320 Loss of function	AR or digenic	
Familial Glucocorticoid deficiency	PRDX3	604769 Loss of function	Possible Digenic	
Familial Glucocorticoid deficiency, type 5	TXNRD2	606448 Loss of function	AR	Possible structural cardiac abnormalities
Defects in Metabolic pathways				
Wolman disease and Cholesteryl ester storage disease	LIPA	613497 Loss of function	AR	Malabsorption, gastrointestinal symptoms, liver dysfunction, atherosclerosis, serum lipid abnormalities, hypersplenism, xanthelasma, hypercholesterolemia, steatorrhea, enlarged adrenal glands with punctate calcifications
Smih-Lemi Opitz disease	DHCR7	602858 Loss of function	AR	Mental retardation, craniofacial malformations, growth failure, polydactyly, sex reversal, renal hypoplasia, genital abnormalities
Abeta-lipoproteinemia	MTP	157147 Loss of function	AR	Hypocholesterolemia and malabsorption of lipidsoluble vitamins leading to retinal degeneration, neuropathy, and coagulopathy. Hepatic steatosis
Sitosterolemia (phytosterolemia)	ABCG5, ABCG8	210250 Loss of function	AR	Very high levels of plant sterols in the plasma and develop tendon and tuberous xanthomas, accelerated atherosclerosis, and premature coronary artery disease, short stature, gonadal failure
Adrenoleukodystrophy	ABCD1	300371 Loss of function	XLR	Cognitive and behavioural problems, motor problems, visual impairment, headache, seizures, bowel and bladder disturbance, sexual dysfunction, elevated VLCFA's
Peroxisome biogenesis disorder 1A (Zellweger)	PEX1	602136 Loss of function	AR	Congenital malformations, developmental delay, corneal clouding, retinal dystrophy, sensorineural hearing loss, neurological deficits, liver dysfunction, renal oxalate stones, failure to thrive, skeletal abnormalities, urogenital anomalies, liver dysfunction,
Sphingosine-1-phosphate lyase deficiency	SGPL1	603729 Loss of function	AR	Steroid resistant nephrotic syndrome, Ichthyosis, primary hypothyroidism, hypogonadism, cryptorchidism and immune and neurological abnormalities
Kearns–Sayre syndrome	mtDNA deletion	530000 mitDNA deletion	IC/mit	Ophthalmoplegia, pigmented retinopathy, cardiomyopathy, cerebellar ataxia, endocrinopathies, hypoparathyroidism, type 1 diabetes, rarely AI
Refsum disease	PHYH	266500 Loss of function	AR	Retinitis pigmentosa, peripheral neuropathy, cerebellar ataxia, ichthyosis and elevated protein levels in the cerebrospinal fluid (CSF) without an increase in the number of cells
DNA replication and repair disorders				
Immunodeficiency 54,	MCM4	602638 Loss of function	AR	Severe intra and extra uterine growth retardation, microcephaly, decreased numbers of natural killer cells, and recurrent viral infections, hepatosplenomegaly, hyperpigmentation, increased susceptibility to lymphoproliferative disorders, chromosomal breakage
Achalasia-addisonianism-alacrimia syndrome	AAAS	605378 Loss of function	AR	Microcephaly, short stature, neurological symptoms, alacrimia, autonomic dysfunction, developmental delay, hyperkeratosis, achalasia, optic atrophy
Monogenic causes of autoimmune adrenalitis				
Autoimmune polyendocrinopathy syndrome, type I	AIRE	607358 Loss of function/DominAaRnt/ AnDegative	Ae/ AD	Candidiasis, hypoparathyroidism, insulin dependent diabetes, alopecia, vitiligo, gastrointestinal complaints, eye abnormalities
Conditions associated with adrenal dysgenesis				
Congenital adrenal hypoplasia	NROB1 (DAX1)	300473 Loss of function	XLR	Genital abnormalities, hypogonadotrophic hypogonadism, occasionally gonadotrophin independent precocious puberty, Duchenne muscular dystrophy + glycerol kinase deficiency (psychomotor retardation) for Xp21 contiguous gene syndrome
IMAGe syndrome	CDKN1C	600856 Gain	AD—maternal	Intrauterine growth restriction, metaphyseal dysplasia, adrenal hypoplasia congenita, hypogonadotrophic hypogonadism, and genitourinary abnormalities in males, facial dysmorphism
MIRAGE syndrome	SAMD9	610456 Gain	AD	Myelodysplasia, infection, restriction of growth, adrenal hypoplasia, genital phenotypes, and enteropathy, cognitive impairment, hypoplastic thymus, hyperpigmentation, skeletal anomalies, gastrointestinal problems,
Steroidogenic factor 1 deficiency	NR5A1	184757 Loss of function	AD	46,XY DSD, gonadal insufficiency, 46,XX Sex Reversal, Premature ovarian failure, Spermatogenic failure

**Table 3:** Genetic causes of secondary adrenal insufficiency

Disorder	Gene	OMIM	Mechanism of action	Inheritance	Additional phenotypic features
Pituitary development					
Combined pituitary hormone deficiency	Prophet of Pit-1 (PROP1)	601538	Loss of function	AR	Progressive development of CPHD in the order GH, PRL, TSH, LH/FSH, (ACTH— late onset); anterior pituitary may be hypoplastic, normal or enlarged
	Homeobox gene 1 (HESX1)	601802	Loss of function	AR/AD	CPHD + optic nerve hypoplasia and midline brain defects/agenesis of corpus callosum (= septo-optic dysplasia); anterior pituitary hypoplastic or ectopic; autosomal recessive, autosomal dominant
	LIM homeobox 3 (LHX3)		Loss of function	AR	CPHD with involvement of GH, TSH, gonadotrophins, PRLs; ACTH may be deficient; limited neck rotation, short cervical spine, sensorineural deafness; anterior pituitary hypoplastic, normal or enlarged
	LIM homeobox 4 (LHX4)	602146	Loss of function	AD	CPHD with involvement of GH, thyrotropin, and ACTH secretion, cerebellar abnormalities; anterior pituitary hypoplastic or ectopic
	SRY-box 3 (SOX3)	313430	Loss of function	XLR	Infundibular hypoplasia, CPHD, variable: mental retardation
POMC synthesis and processing					
POMC deficiency	Proopiomelanocortin (POMC)	176830	Loss of function	AR	Hyperphagia, early onset obesity, cholestasis, pigmentary defect, central hypothyroidism, GH deficiency, hypogondotrophic hypogonadism
Proprotein convertase 1/3 deficiency	Proprotein Convertase Subtilisin/Kexin Type 1(PCSK1)	162150	Loss of function	AR	Obesity, gastrointestinal disturbance, other pituitary deficiencies possible, elevated proinsulin, low insulin
POMC lineage differentiation and transcription defects					
Congenital isolated ACTH deficiency	Tpit or T-box 19 (TBX19)	604614	Loss of function	AR	Neonatal presentation
Other syndromes associated with secondary adrenal insufficiency					
Prader Willi syndrome	Imprinted gene cluster 15q11.2		Loss of function	IC	syndrome of hypotonia, short stature, hyperphagia, obesity, hypogonadism, psychomotor delay, and sleep-related breathing disorders
Pallister Hall syndrome	(Including genes SNRPN, NDN) GLI3	176270 165240	Loss of function	AD	Hypothalamic hamartomas, hypopituitarism, imperforate anus and postaxial polydactyly

## Defects in ACTH signalling

### Familial glucocorticoid deficiency

Familial glucocorticoid deficiency (FGD) is a rare autosomal recessive endocrine disorder, caused by the inability of the adrenal cortex to generate a physiological response to ACTH stimulation, with consequent impaired cortisol production and, therefore, resulting in AI [3–5]. The exact worldwide incidence and prevalence of FGD are currently unknown, with the exception of a specific population, the Irish Travelers, in which a prevalence of 1:200,000 cases was reported [6, 7].

FGD is a genetically heterogeneous disease, as up to seven different genes, including *MC2R*, *MRAP*, *NNT*, *STAR*, *MCM4*, *TXNRD2*, and *SGPL1*, have been reported to be involved in its pathogenesis [4, 5, 8–10]. The most frequent genetic alterations are represented by the inactivating variants of *MC2R*, encoding the melanocortin receptor 2 (MC2R), occurring in 25% of cases and related to the FGD type 1 variant, and *MRAP*, encoding the melanocortin receptor 2 accessory protein (MRAP), occurring in 20% of cases and related to the FGD type 2 variant [5, 11–14]. Less frequently, FGD is caused by inactivating variants of *NNT*, encoding the nicotinamide nucleotide transhydrogenase (NNT) (10% of cases) [15], *STAR*, encoding steroidogenic acute regulatory protein (StAR) (5–10% of cases) [16], *MCM4*, encoding the mini-chromosome maintenance complex component 4 (MCM4) (< 5% of cases) [7], *TXNRD2*, encoding thioredoxin reductase 2 (TXNRD2) (< 5% of cases) [17], and *SGPL1*, encoding sphingosine-1-phosphate lyase 1 (SGPL1) (< 5% of cases) [5]. Classically, FGD is characterized by isolated deficiency of cortisol with normal production of aldosterone. This is certainly the case for Type 1 and Type 2 FGD although as more genes are identified MC deficiency is sometimes associated such as in NNT-related FGD.

Clinical features of FGD mainly include AI, skin hyperpigmentation, recurrent hypoglycemia, seizures, and failure to thrive, although some specific features may be observed according to the underlying causes of the disease [4, 5].

### Type 1 and Type 2 FGD

In healthy subjects, MC2R is the principal adrenal ACTH-binding receptor and MRAP is a trans-membrane accessory protein responsible for the correct MC2R trafficking from the endoplasmic reticulum to the cell surface and to allow for signalling [14]. In type 1 and 2 FGD patients, *MC2R* and *MRAP* variants induce ineffective ACTH-binding, impaired signal transduction or deficient MC2R trafficking to the cell surface, leading therefore to ACTH unresponsiveness and PAI [14]. Although sharing similar pathogenesis, patients with type 1 and 2 FGD present with different phenotypes, as the type 1 FGD is associated with early childhood onset and tall stature, whereas type 2 FGD is associated with infancy onset and normal height [18, 19].

## Alterations in antioxidant mechanisms

### NNT-related FGD

In healthy subjects, NNT is an integral protein of the inner mitochondrial membrane, responsible for nicotinamide adenine dinucleotide phosphate hydrogen (NADPH) production necessary for glutathione-related mitochondrial detoxification processes [20]. In FDG patients, *NNT* variants induce an increase in reactive oxygen species and consequent oxidative damage to mitochondria, with a direct negative impact on adrenal steroidogenesis and, therefore, PAI [5, 15]. Currently, the mechanisms behind the specific NNT-related adrenocortical cell damage have not been clarified; however, evidence from NNT-deficient mice models have shown a markedly decreased expression of the adrenal steroidogenic enzyme Cyp11a1, probably related to the excessive oxidative damage induced by NNT loss of function [15]. NNT-related FGD has an early childhood onset, and, unlike the remaining forms of FGD, it has been also reported to be associated with MC deficiency in about 17% of cases [21]. Moreover, as *NNT* variants, and particularly heterozygous loss of function variants, have been associated with left ventricular noncompaction [22], cardiac alterations have also been rarely reported in FGD patients [23].

### StAR-related FGD

In healthy subjects, StAR is a steroidogenic enzyme involved in cholesterol transport across mitochondrial membranes, a crucial step in steroidogenesis in both the adrenal glands and gonads [24]. Usually, StAR variants are associated to lipoid congenital adrenal hyperplasia (LCAH), in which the alteration of the StAR-dependent cholesterol import deeply impairs cortisol, aldosterone, and androgen production, both in the adrenal gland and in gonadal steroidogenesis, leading to AI and, in males, to lack of normal virilization of external genitalia [24]. However, a partial loss of function of StAR has been also observed in FGD patients with normal genitalia, thus not being initially addressed as lipoid CAH (LCAH) patients [16]. Currently, StAR-related FGD patients are still considered as FGD patients, although the clinical phenotype and the molecular pathogenesis is gradually leading towards the inclusion of these patients in the definition of “non-classical” LCAH [16, 24, 25].

### MCM4-related FGD (“Irish Travelers FGD”)

In healthy subjects, MCM4 is part of a complex genetic machinery responsible for genome stability and correct DNA replication [26]. In FGD patients, the specific impact of *MCM4* variants is not fully understood; however, mouse models have shown a marked decrease in steroidogenic cells in adrenal zona fasciculata, thus suggesting a potential pathogenetic mechanism for MCM4-related FGD [7]. Currently, MCM4-related FGD has been identified only in a specific population, the Irish Travelers, with a specific phenotype, characterized by gradual onset AI, natural killer cell deficiency with recurrent infections, and growth retardation [7]. The gradual onset of AI seems to be related to the partially preserved function of mutated MCM4, being able to maintain normal adrenal function in infancy and early childhood [7].

### TXNRD2-related FGD

In healthy subjects, TXNRD2 is a mitochondrial protein responsible for the maintenance of thioredoxin in a reduced state, allowing therefore a correct mitochondrial superoxides disposal [27]. In FGD patients, *TXNRD2* variants lead to excessive levels of mitochondrial superoxides, only partially managed by the glutathione system, thus inducing adrenal gland dysfunction; however, the exact mechanism behind TXNRD2-related adrenal damage is currently unknown [17]. Currently, TXNRD2-related FGD has been identified only in young members of a Kashmiri family, that were mainly asymptomatic at the time of testing, ranging from infancy to early adolescence [17]. Moreover, although *TXNRD2* variants have been associated with lethal cardiac developmental abnormalities in mouse models [22], only one patient presented cardiac defects leading to heart failure [17].

### SGPL1-related FGD

In healthy subjects, SGPL1 is involved in the catabolism of sphingosine-1-phosphate (S1P), a key element of G protein-related intracellular signaling, with a prevalent pro-proliferative and anti-apoptotic action, including a pro-secretive effect on adrenal glands [5, 28]. In FGD patients, *SGPL1* variants lead to a pathological accumulation of S1P intermediates, including ceramides, which are known to be steroidogenesis inhibitors, thus overcoming S1P pro-secretive effects on cortisol production and therefore leading to AI [5]. SGPL1-related FGD is associated with congenital steroid-resistant nephrotic syndrome, whereas other clinical features, including primary hypothyroidism, ichthyosis, neurodevelopmental delay, lymphopenia, and dyslipidaemia, are less frequently observed [29]. However, cases of SGPL1-related FGD without nephrotic syndrome have also been reported, although rarely [30].

## Defects in metabolic pathways

### Adrenoleukodystrophy

Adrenoleukodystrophy (X-ALD) is a rare X-linked disease of peroxisomal oxidation caused by variants in the *ABCD1* gene [31], encoding for an ATP-binding cassette transporter for very long chain fatty acids (VLCFA) that mediates the influx into peroxisomes for β-oxidation, with more than 2500 known variants [32]. Classically, X-ALD incidence was reported to have an initial incidence of 1:21,000 births in males and 1:16,800 in females [33], but recent evidence from the introduction of newborn screening for X-ALD reported a higher incidence of 1:3878 births in males and 1:4845 births in females [34, 35]. Males generally have a more severe phenotype. The pathogenetic trigger of X-ALD is the accumulation of very long chain fatty acids (VLCFA, C ≥ 22:0), particularly hexacosanoic acids (C26:0) and lignoceric acids (C24:0), in target cells. Preferential sites of accumulation are the adrenal zona fasciculata and zona reticularis, testicular Leydig cells, and Schwann cells [33]. The mechanism for cellular damage is still under debate, and could be due to direct cytotoxicity, oxidative stress and, in the adrenal, direct damage to plasma membrane interfering with binding of ACTH to its receptor, resulting in a form of ACTH resistance [34].

Although its clinical presentation is very variable, the most common features are represented by PAI and demyelination, occurring both in the central and peripheral nervous systems [32]. Affected males usually develop signs and symptoms of PAI due to progressive adrenal damage during childhood, with the most common age of onset between 3 and 10 years [32, 35]. Heterozygous women with X-ALD have a wide spectrum of clinical manifestations, ranging from mild to severe phenotypes [36]. Prevalence of AI in patients with X-ALD is estimated around 70% [32], and median delay to diagnosis was 3.5 years [32]. MC deficiency is rarer compared to GC deficiency, with 57% of X-ALD patients with AI requiring MC replacement [32]. As for central nervous system involvement, the natural history of cerebral ALD in children is characterized by progressive cerebral demyelination, with functional decline to a vegetative state within 2–5 years, and a slower progression in adolescents. In adults, cerebral ALD is usually characterized by focal neurological deficits, dementia, and behavioral disturbances [35]. Adrenomyeloneuropathy (AMN) develops due to spinal cord injury, with spastic paraparesis and sensory ataxia, usually worsening and requiring the patients to be wheelchair-bound in their 6^th^ decade of life. AMN coexists with cerebral damage in 63% of cases [35]. Patients with X-ALD often also present with testicular dysfunction due to accumulation of VLFA in Leydig cells, leading to primary hypogonadism. No clear genotype–phenotype correlation has been discovered yet.

#### Allogenic stem cell transplantation and lentiviral gene therapy

Two relatively new types of therapy are currently being appraised in adults with cerebral ALD, namely allogenic stem cell transplantation (ASCT) and lentiviral gene therapy. The results have recently been extensively reviewed [37]. Patients with relatively mild demyelination scores showed effective lack of progression of neurological symptoms after ASCT, whereas those with advanced disease showed a poorer response, emphasizing the necessity of early identification of cerebral disease [37]. The mechanism for improvement of neurological symptoms after ASCT has not been clearly explained. Lentiviral gene therapy has shown positive results in a small number of patients and is still in the process of evaluation [37]. The comparison of the results of gene therapy versus ASCT is complex and in terms of patient management is beyond the scope of this review. Both therapies remain experimental, but are options for the control of cerebral ALD.

## DNA replication and repair disorder

### Allgrove syndrome/Triple A syndrome

Allgrove syndrome, also called Triple A syndrome (TAS), is an autosomal recessive disorder characterized by the triad achalasia, PAI, and alacrimia. TAS is a rare disease, with less than 50 described families characterized by AAAS variants in 90% of patients. More recently, several cases of late-onset forms of the disease are slightly expanding the number of diagnosed patients [38].

TAS is mainly caused by variants in the AAAS gene, encoding the protein ALADIN, a nucleoporin scaffold protein of the WD repeat family. It is involved in many different functions such as protein–protein interaction, cytoskeleton assembly, control of cell division, RNA processing and DNA repair [39]. The *AAAS* gene localization is 12q13, with 16 exons encoding for the 546 aminoacid protein ALADIN, mostly expressed in pancreas, adrenal and pituitary gland [39, 40]. The pathogenetic mechanisms of TAS are still largely unclear, and no clear genotype–phenotype correlation has been identified yet. However, an increased susceptibility to oxidative stress has been described in *AAAS* mutated cells, increasing cell death, and possibly explaining the progressive nature of the disease [41]. The effects of *AAAS* variants on adrenal cells may also involve the interactions of ALADIN with Progesteron Receptor Membrane Component 2 (PGRMC2), a protein of the membrane- associated progesterone receptor (MAPR) family that can regulate the activity of CYP450 cytochromes [42]. Thus, by altering PGRMC2 localization, defective ALADIN protein might lead to adrenal atrophy and altered steroidogenesis [42]. The last revision of published literature has recorded 74 variants in the *AAAS* gene [43].

The majority of patients with TAS show alacrimia as the presenting symptom, affecting 90–100% of patients and usually present during the first year of life. However, it is often overlooked until symptoms of achalasia, including vomiting, weight loss, difficulty to swallow, and coughing, occur [43]. Esophageal achalasia of cardias is present in 75–85% of TAS patients [43] in most cohorts, and its onset is between three months and 16 years of age [43]. PAI should be investigated in all infants and pediatric patients with achalasia and alacrimia since it can lead to life-threatening adrenal crises or severe hypoglycemia. PAI onset is usually within the first 10 years of life [43], and signs and symptoms of PAI, including hyperpigmentation, hypotension, hypoglycemia, and hyponatremia, are usually developed way before diagnosis. Adrenal androgen secretion is often impaired in TAS, whereas MC secretion is preserved.

Beyond the classic triad, patients may also develop neurological manifestations, including motor neuron neuropathy, motor-sensory or autonomic neuropathy, Parkinsonism, optic atrophy, cerebellar ataxia, and even mild dementia [44]. Age of onset of neurological disorders is usually later than the classic triad, between 2 and 25 years of age [43]. Autonomic dysfunction is the most prevalent form of neurological damage in TAS, followed by neurogenic bladder, ataxia, dysarthria, distal motor and sensory neuropathy and disorders of movement and intellectual disability [43].

Cases of TAS with adult onset have been described, and patients often show incomplete presentation with neurological symptoms as leading features [38].

## Autoimmune Addison’s Disease

Autoimmune Addison’s Disease (AAD) has been described since the 1950s in association with other autoimmune endocrine diseases, configuring autoimmune polyglandular syndromes (APS). The estimated prevalence of APS-1 is 1:100,000 in most countries, while some regions, such as Finland and Sardinia show a higher prevalence reaching 1:25,000 and 1:19,000, respectively [45]. In the 1980s, the segregation of AAD in at least two modalities in the context of APS identified two different patterns of inheritance[46], leading to the distinction between type 1 and type 2 APS. Due to its earlier onset and higher prevalence in childhood, this review will focus on type 1 APS (APS-1).

APS-1 is a rare autosomal recessive disease, also known as autoimmune-polyendocrinopathy-candidiasis-ectodermal dystrophy (APECED), caused by variants in the autoimmune regulator gene (*AIRE*) [47], which is expressed in thymic medullary epithelial cells and dendritic cells. The physiological role of *AIRE* is to mediate the ectopic expression of tissue-restricted proteins, allowing negative selection of autoreactive thymocytes and improving self-tolerance. *AIRE* is located in the 21q22.3 region and contains 14 exons encoding a 545-amino-acid protein with a molecular weight of 57.5 kDa [48]. The major domains in the AIRE protein are the caspase recruitment domain/homogeneously staining (CARD/HSR) region, a SAND (SP100, AIRE, Nuc p41/75, DEAF) domain, four LXXLL (L is leucine and X is any amino acid) motifs, and two plant homeodomain (PHD) zinc fingers. More than 100 variants have been described and exons 6 and 8, corresponding to CARD and PHD domains, are most frequently affected. Most variants are inherited in an autosomal-recessive pattern in the “classical” APS-1 phenotype. In a recent meta-analysis of APS-1 patients, p.R257* and p.L323Sfs*51 were the most common variants [45].

In its “classical” form, APS-1 is characterized by the triad of hypoparathyroidism, PAI, and chronic mucocutaneous candidiasis [49], with the latter usually being the first presenting conditions and the former the first presenting autoimmunity [45, 50]. However, recent studies have expanded its phenotypic landscape, showing that patients can develop more than 20 other organ-specific minor immune-related conditions, the most frequent being represented by primary ovarian insufficiency, enamel hypoplasia, and autoimmune enteropathy [50]. More rarely, patients can develop other autoimmune processes targeting non-endocrine organs, including keratitis, hepatitis, nephritis, pancreatitis, and functional asplenia [50]. Nevertheless, APS-1 diagnosis occurs in the presence of at least two of three classic symptoms, regardless of concomitant immune diseases [51]. Conversely, the autosomal dominant “non classical” form is characterized by later onset and more variable presentations [52, 53].

According to a recent systematic review, the median age of testing for APS-1 was 9.0 years (interquartile range 5.0–17.0) with a median diagnostic delay of 5.0 years [45]. Moreover, the median age of presentation of the first, second and third autoimmune diseases were 5.0 (3.0–8.0), 8.8 (5.6–12), and 11.0 (8.0–17.0) years, respectively.

The clinical picture of APS-1 is very variable even among identical genotypes. Other influencing factors such as environmental, genetic, and stochastic events are probably involved in the presenting phenotype [48]. Even though several correlations between specific variants and the severity of the phenotype have been proposed, they are yet to be confirmed [53]. More specifically, the only apparently constant genotype–phenotype correlation is the protective effect of the pY85C variant against PAI and chronic mucocutaneous candidiasis [54] and the increased severity of phenotype in patients with nonsense and/or frameshift insertion-deletion variants [45]. Other small studies have shown that in Scandinavian APS-1 patients, PAI seems to be associated with HLA-DRB1*03, alopecia with HLA-DRB1*04-DQB1*0302, and that HLA-DRB1*15-DQB1*0602 is apparently protective against type 1 diabetes [55].

Recently, Oftedal and colleagues [52] have identified specific variants, mostly clustered in the PHD1 finger domain [45, 52], corresponding to variable phenotypes of autoimmune endocrine diseases with dominant inheritance. Since this “non-classical” presentation due to AIRE heterozygous variants and monogenic autoimmunity often does not meet diagnostic criteria for APS-1, it is possible that the prevalence of this disease is largely underestimated.

## Conditions associated with adrenal dysgenesis

### Congenital adrenal hypoplasia

Congenital adrenal hypoplasia (adrenal hypoplasia congenita, AHC) is a rare condition characterized by an impaired development of the adrenal glands, caused by nuclear receptor variants that regulate both adrenal and reproductive function (DAX1 and Sf-1 variants) or multisystem intrauterine growth restriction (IUGR) syndromes.

### X-linked AHC

The most common cause of AHC is the X-linked form. Being an X-linked disease, AHC primarily affects boys, although some cases are also reported in girls due to skewed X-inactivation [56–58].

X-linked AHC is caused by a germline variant of the nuclear receptor protein DAX-1, encoded by the gene *NR0B1* located on the short arm of the X-chromosome. DAX-1 protein is expressed in tissues involved in steroid hormone production at multiple levels, from the hypothalamus and the pituitary to the adrenal cortex and the gonads [57, 59, 60]. More than 200 different *NR0B1*/DAX1 gene deletions or variants have been identified in patients suffering from PAI and reproductive disorders, showing an extreme variability in terms of onset, disease severity, and clinical phenotypes [61]. As even intrafamilial clinical heterogeneity was reported, no clear genotype–phenotype correlation was observed, leading some authors to hypothesize epigenetic and non-genetic mechanisms in the definition of patients’ phenotypes [60, 61]. A detailed family history and a follow-up with a genetic counselor are crucial for preventing life-threatening adrenal crises in close male relatives [60, 61].

Although clinically heterogeneous, the classic clinical features of X‐linked AHC generally include PAI, hypogonadotropic hypogonadism (HH), and infertility [59, 62]. PAI is usually observed in the first two months of life, presenting as salt-wasting adrenal crisis in patients with normal external genitalia [63]. More insidiously, this condition can be diagnosed later in childhood due to variable GC and MC deficiency, in adolescence due to HH and delayed puberty, or even in adulthood due to infertility. In some patients with AHC, the apparent MC deficiency frequently precedes GC deficiency [59, 60, 64]. External genitalia often appear normal in the neonatal period. Although AHC is commonly associated with HH, precocious puberty was paradoxically reported in a subgroup of AHC patients [61, 63]. Moreover, a single case of short stature and growth hormone deficiency has been reported [63].

### Intrauterine growth retardation (IUGR) syndromes associated with PAI

IUGR syndromes associated with PAI are rare and a rapid and correct diagnosis is challenging due to severity and overlapping of the extra-adrenal features. The most common IUGR syndromes showing adrenal hypoplasia are IMAGe, IMAGe-like, and MIRAGE syndromes. IMAGe syndrome is characterized by intrauterine growth restriction, metaphyseal dysplasia, adrenal hypoplasia, genitourinary anomalies, caused by gain-of-function variants of the cell-cycle repressor *CDKN1C* [65], whereas IMAGe-like syndrome also includes immune dysfunction of variable severity and is caused by autosomal recessive loss‐of‐function variants of polymerase epsilon‐1 (POLE1). MIRAGE syndrome is characterized by myelodysplasia, infections, restriction of growth, adrenal hypoplasia, genital phenotypes, and enteropathy, and is caused by a heterozygous gain of function variant in the growth repressor sterile alpha domain containing 9 gene (SAMD9); although usually diagnosed at birth, in some MIRAGE syndrome patients PAI onset occurred in early infancy [66].

### Rare monogenic syndromes associated with PAI

Other known rare syndromic monogenetic causes of adrenal dysgenesis are SERKAL syndrome characterized by sex reversal with dysgenesis of kidney and lungs, due to *WNT4* mutation, Pallister-Hall syndrome, characterized by hypothalamic hamartoma, central polydactyly, and visceral malformations, due to *GLI3* variants, Pena-Shokeir syndrome type I, characterized by fetal akinesia, IUGR, joint contractures, short umbilical cord, and pulmonary hypoplasia, due to *DOK7* or *RAPSN* variants, pseudotrisomy 13, characterized by holoprosencephaly, severe facial anomalies, postaxial polydactyly, and various congenital defects, Galloway–Mowat syndrome, characterized by microcephaly, developmental delay, early-onset intractable epilepsy, and renal alterations, due to *WDR73* variants, and Meckel–Gruber syndrome, characterized by renal cystic dysplasia, occipital encephalocele, postaxial polydactyly, hepatic developmental defects, and pulmonary hypoplasia, due to *MKS1* variants [51, 67]. All these syndromes have poor prognoses.

### Pathogenic variants of SF-1

Steroidogenic factor 1 (SF-1) is a nuclear receptor encoded by the *NR5A1* gene that regulates both adrenal and reproductive development [57]. Approximately 250 patients with pathogenic variants in *SF‐1/NR5A1* have been described, with a large spectrum of phenotypes, mostly represented by 46, XY subjects with testicular dysgenesis, showing a female phenotype. A male phenotype with infertility and adrenal impairment has also been described, as have 46, XX women with ovarian insufficiency [57]. Ectopic spleen or spleen agenesis were also reported [62].

## Conditions associated with isolated mineralocorticoid deficiency

### Type 1 pseudohypoaldosteronism

Type 1 pseudohypoaldosteronism (PHA1) is a group of rare genetic diseases caused by non-responsiveness of target tissues to aldosterone. Currently, two types of PHA1 are described: renal PHA1, or type a PHA1 (PHA1a), and systemic PHA1, or type b PHA1 (PHA1b) [68]. PHA1a is an autosomal dominant disease, caused by dysfunctional or non-functioning mineralocorticoid receptor (MR) expression in the kidney, leading to renal aldosterone resistance, whereas PHA1b is an autosomal recessive disease, caused by defective epithelial sodium channel (EnaC) expression in different tissues, including salivary and sweat glands, lung, kidney, and colon, leading to systemic aldosterone resistance [69, 70].

#### Pseudohypoaldosteronism type 1a PHA1a

PHA1a is caused by variants in *NR3C2* gene, coding for the MR, mainly represented by frameshift and nonsense variants leading to stop codons and consequent non-functioning or malfunctioning MR, although also missense variants have been recently reported. Interestingly, a homozygous missense variant of the aldosterone binding domain has been reported to have a dominant negative effect on wild type MR function, whereas variants of the DNA binding domain, leading to a reduced activation of the MR, had no negative dominant effect [71]. Moreover, a chromosomic disruption leading to a balanced paracentric inversion with karyotype 46XX 4 inv (q31.2q35) causing PHA1a has been described in a patient without mutations in his relatives [68]. Variants were found in all exons and there is currently no certain mutational hot spot. However, in spite of this, a possible hot spot for loss of function variants has been suggested, as recurrence of the same variant in the exome coding for the C-terminal ligand binding domain of the MR was observed in some unrelated families with PHA1a [69].

The clinical presentation of PHA1a is highly variable. Newborns may develop symptoms from first days (5–7) to 20 days of life [70], although also rare cases of onset during the second month of life have been described [72]. Even if cases of completely asymptomatic PHA1a cases were reported, it is usually associated with mild dehydration, vomiting, and failure to thrive [70, 73]]. Less frequently, PHA1a leads to severe dehydration, hyperkalemia with potential cardiac arrhythmias, metabolic acidosis, and shock [70, 71]. Beyond hyperkalemia and metabolic acidosis, also hyponatremia and elevated renin and aldosterone levels may be observed [70, 73]. Moreover, a higher risk of preterm birth and small for gestational age newborns was reported [68].

Sometimes, infants with congenital urinary malformations may develop symptoms of urinary tract infections together with a transient PHA1, called secondary PHA1. Interestingly, a deletion of exons 3–5 in NR3C2 has been described in a patient with suspected secondary PHA1 in the presence of urinary infections, posterior urethral valves, severe vesicoureteral reflux, and high parental plasma aldosterone levels, in the presence of a correct molecular diagnosis of PHA1a. Together with the phenotypic heterogeneity, these data suggest that many infants, including those with secondary PHA1, may be actually undiagnosed cases of PHA1a [74].

Over time, PHA1a often progresses towards a spontaneous improvement, that may even reach complete clinical and biochemical resolution within the first years of life. Progressive renal maturation during the first months of life, leading to increased sensitivity of MRs to aldosterone and consequent compensation to MRs haploinsufficiency, has been advocated as a potential explanation for the PHA1a spontaneous improvement during childhood [71].

Currently, no clear genotype–phenotype correlation has been observed, as it is commonly reported that parents not experiencing clinical symptoms or experiencing mild symptoms with spontaneous resolution and not requiring treatment gave birth to children experiencing a severe phenotype, although the same variants were observed [70, 72]. Whereas elevated aldosterone levels remain as a biochemical marker of PHA1a in these patients during adulthood is controversial [72].

#### Pseudohypoaldosteronism type 1b PHA1b

PHA1b is caused by variants in the *SCNN1A*, *SCNN1B*, and *SCNN1G* genes, coding for the alpha, beta, and gamma subunits of ENaC, respectively, and leading to dysfunctional channel activity. SCNN1A missense variants and deletions are the most frequent cause of PHA1b and are mainly recognized in homozygous patients [70], although SCNN1B and SCNN1C frameshift deletions have also been recognized as responsible for PHA1b[70, 75]. Moreover, a potential founder variant in Arab populations, the *SCNN1A* p.I68Tfs*76 variant, was recently found in unrelated families [76].

As PHA1b is caused by a systemic dysfunction of enac, multi-organ water and salt loss is observed since the first days of life, leading to severe dehydration, resistant electrolyte imbalance with potential cardiac arrhythmias and cardiac arrest, impaired lung fluid re-absorption with consequent neonatal respiratory distress syndrome and respiratory infections, increased sweat sodium levels with consequent dermatitis and cutaneous infections, and gastrointestinal motility alteration with consequent vomiting and poor feeding. Altogether, these alterations may result in failure to thrive or growth delay [77]. As for PHA1a, spontaneous improvement during childhood can be also observed in PHA1b. Increased Sodium Chloride Cotransporter (NCC) thiazide sensitive channel activation in the proximal nephron and potential initially non-functional enac channel maturation during childhood have been advocated as potential explanations [71].

Systemic, severe PHA1b is the most frequent form of this disease, but milder clinical features, with no pulmonary involvement or showing exclusive sweat salt loss, without systemic complications, have also been reported [78].

Although a clear genotype–phenotype correlation is not reported, in patients carrying *SCNN1A* variants, lung involvement is usually not observed [70, 71], and particularly patients carrying missense variants have shown milder clinical pictures with spontaneous improvement during childhood [76]. Also, lower aldosterone levels have been observed in patients with missense variants of *SCNN1A* or *SCNN1B*, likely due to partially maintained function of enac, although they are not necessarily associated with a less severe clinical picture [70]. Recently, the association of nephropathy with the typical clinical manifestations of PHA1b was observed in an infant with a new homozygous *SCNN1C* variant, whose parents were both heterozygotes and had no kidney disease. This variant has been found to be responsible for renal damage due to pro-apoptotic, inflammatory, and oxidative action [75].

### Aldosterone synthase deficiency

Primary hypoaldosteronism due to aldosterone synthase deficiency (ASD) is a rare congenital disorder due to dysfunctional aldosterone synthase, responsible for the last steps of the synthesis of aldosterone. Aldosterone synthase catalyzes the 18-hydroxylation of corticosterone to form 18OH-corticosterone, and finally the 18-oxydation of 18OH-corticosterone to form aldosterone.

According to the alteration in aldosterone synthase activity, two forms of ASD may be identified, type 1 (ASD1) and type 2 ASD (ASD2). In ASD1, both hydroxylation and oxidation processes are impaired, whereas in ASD2 only oxidation process is impaired.

Aldosterone synthase is coded by the *CYP11B2* gene, with nonsense and missense variants representing the most frequent cause of ASD [79]. In ASD1 patients, *CYP11B2* variants cause complete enzyme inactivation, compromising heme-binding sites or highly conserved sites necessary for enzymatic activity, and mainly homozygous genotypes are observed, whereas in ASD2 patients *CYP11B2* variants only cause partial enzyme inactivation and more complex genotypes, including double homozygosis, compound heterozygosis, and triple variants, may be observed [80]. Moreover, a possible founder effect may be observed in some populations, including Turkish patients, in whom a homozygous variant c.788 T > A at codon 263 combined with other mutations in homozygosis was exclusively observed, and patients from Greece, Albany, and Italy, in 95% of whom a variant c.544C > T at codon 185 in homozygosis was observed [79, 81]. ASD clinical symptoms appear within the first 3–4 months of life, usually in the first days or weeks of life, with dehydration, hyponatremia, hyperkalemia, vomiting, failure to thrive, and sometimes metabolic acidosis [80, 82, 83]. Albeit expected, no clinical differences between ASD1 and ASD2 are currently reported, whereas biochemical differences are more clearly evident. In both forms, elevated corticosterone levels are present, but in ASD1 decreased 18OH-corticosterone and aldosterone levels and elevated renin levels may also be observed, whereas in ASD2 elevated 18OH-corticosterone levels and less markedly decreased aldosterone levels and increased renin levels may be observed [80, 82, 83]. Nevertheless, single case reports of patients experiencing only failure to thrive at clinical presentation and no electrolyte disturbances at examination [83] or experiencing spontaneous resolution within 3 months of life [84] are actually reported, suggesting potential, yet unknown, milder disease forms. Moreover, neurosensory deafness has also been described an association with ASD, maybe related to a potential role of aldosterone in the electrolytic homeostasis of endolymph, although the potential role of CYP11B2 is still unclear [84].

Currently, genotype–phenotype correlations are not clearly reported, and many data seem to suggest that the distinction between ASD1 and ASD2 may be blurred. In a single case report a patient carrying a compound heterozygous variant leading to two stop codons of *CYP11B2* and therefore to potential complete enzyme inactivation and ASD1 experienced a biochemical profile that was intermediate between ASD 1 and 2 [80]. Therefore, some authors suggest that a distinction between ASD1 and ASD2 should no longer be made, and that other genes should be identified to explain the previously observed biochemical differences [80, 83].

## Diagnosis of rare forms of adrenal insufficiency

Although clinically and biochemically heterogenous, all rare forms of pediatric AI include GC and/or MC deficiency as their main clinical feature, therefore a formal diagnosis is required before further investigation in terms of comorbidities and genetic causes.

### Glucocorticoid deficiency

A basal early morning blood test for cortisol, ACTH and cortisol circadian rhythm is very useful and an early morning serum cortisol of < 100 nmol/l is often diagnostic of AI and an ACTH test may not be needed. In infants a basal sample during hypoglycaemia is useful for corticotroph status. The gold standard investigation for AI diagnosis is the cosyntropin intravenous stimulation test, at the dosage of 250 mcg in adults and children older than 2 years, 15 mcg/kg in infants and 125 mcg for children younger than 2 years [85]. Peak cortisol at 30 or 60 min ≥ 500 nmol/l excludes AI, even though cut-offs are assay-dependent [86]. The insulin-induced hypoglycaemia test is useful for assessing ACTH secretion and cortisol response which should be greater than 500 nmol/l.

Patients should be tested if presenting with signs and symptoms of AI (hyperpigmentation in PAI, hyponatremia, hypotension or postural dizziness, nausea and abdominal discomfort, weight loss, fatigue). In children, clinical presentation is even more insidious, including other nonspecific signs such as failure to thrive, dehydration and hypoglycemia. Patients acutely ill with signs or symptoms potentially correlated to AI should undergo baseline evaluation for cortisol and ACTH prior to steroid administration for diagnostic purposes, but prompt treatment should not be further delayed and a cosyntropin test can be performed once the patient is stable [85].

### Mineralocorticoid deficiency

MC deficiency should be suspected in the presence of symptoms of AI. More unspecific symptoms should be expected in infants, considering that in some cases failure to thrive or delayed growth can be the only clinic manifestations of MC deficiency. The diagnosis is supported by hyponatremia, hyperkalemia, dehydration, and occasionally metabolic alkalosis.

In the majority of cases, especially in the context of emergency clinical presentation, the diagnosis of MC deficiency is not the priority, and prompt treatment with GC is started. The presence of isolated MC deficiency or resistance to aldosterone is often suspected when treatment with hydrocortisone is not sufficient to solve dehydration and electrolytic imbalance, or when an ACTH test is performed, and normal cortisol response is proved. [70] In the presence of low aldosterone levels, elevated lying renin, which varies with posture, confirms the diagnosis of aldosterone deficiency. On the other hand, in presence of normal or elevated aldosterone levels and in the absence of cortisol deficiency, normal or low renin levels will not exclude a condition of pseudohypoaldosteronism. When PHA1B is suspected, a sweat test can be performed: high sodium content in sweat is suggestive of PHA1B [70].

### Disease-specific diagnostic assessments

**Fig. 1:**
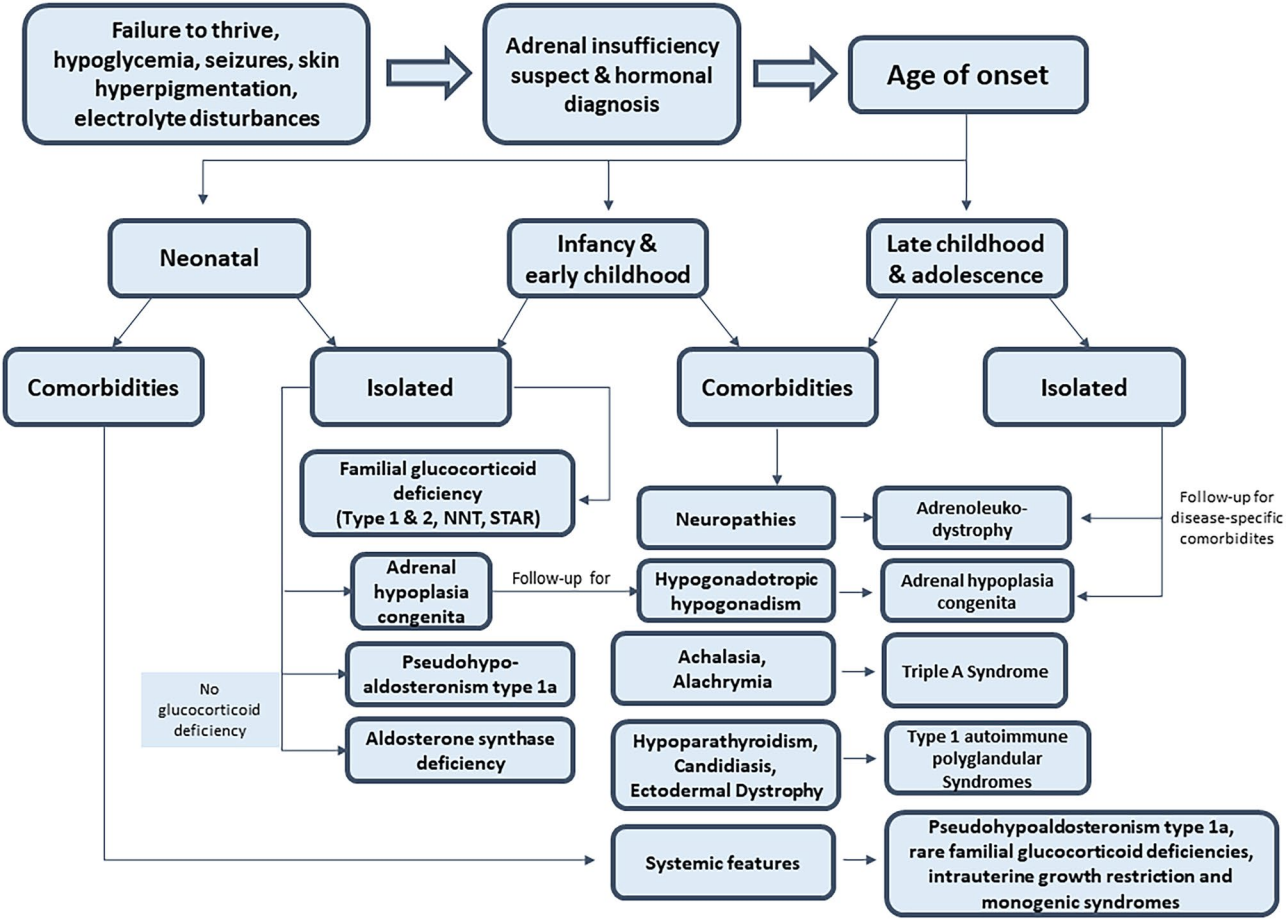
Diagnostic flowchart for paediatric patients with suspected primary adrenal insufficiency (after exclusion of congenital adrenal hyperplasia). NNT: nicotinamide nucleotide transhydrogenase, STAR: steroidogenic acute regulatory protein

Once AI diagnosis is established, further evaluations are necessary to identify the underlying causes in pediatric patients in which congenital adrenal hyperplasia, the most frequent cause, has already been ruled out (Fig. 1). Differential diagnosis should be a stepwise approach, in which clinical features, genetic pedigrees, and disease-specific biochemical markers should be considered. Evaluation of clinical features should include age of onset and eventual comorbidities (Fig. 1).

Considering age of onset, neonatal onset is more common in AHC, PHA1, and ASD, usually presenting with salt-wasting crises, however in AHC, AI is usually the first clinical manifestation. However, a more insidious, gradual onset ranging from infancy to childhood may be observed in APS1, ALD, TAS, and FGD. Notably, X-linked diseases such as AHC and PHA1b may present *de novo*, thus not providing physicians with a positive family pedigree at presentation. AHC and ALD may also present in adolescence, although these patients are usually already followed up for disease-specific disorders, namely HH in AHC patients and neuropathies in ALD patients. However, screening for VLCFA should be done in all male children presenting with primary AI during childhood and adolescence to rule out X-ALD in case where tests for other etiologies (autoimmune, CAH) are inconclusive. X-linked diseases, including AHC and ALD and autosomal recessive disorders, including TAS.

Considering comorbidities, isolated AI is only observed in PHA1a, ASD, NNT and Specific mutation in STAR and type 1 and 2 FGDs, whereas the remaining rare causes of pediatric AI are mainly syndromic. Moreover, disease-specific features, including mucocutaneous candidiasis and primary hypoparathyroidism in APS1, neuropathies in ALD, alacrimia and esophageal atresia in TAS, and multi-system involvement in PHA1b and rare forms of FGDs, are usually already known and diagnosed at AI presentation. This may therefore suggest to physicians to assess GC and/or MC levels before the occurrence of typical AI signs and symptoms. Conversely, in AHC patients AI is usually the first clinical manifestation, so patients should be checked for HH at pubertal age, if not already diagnosed.

Considering genetic pedigrees, they may be available in patients with diseases presenting a well-defined inheritance pattern, including AHC, ALD, TAS, type 1 and 2 FGDs, PHA1, and ASD, in which also parents or other family members may be affected, whereas in diseases with more complex inheritance patterns, as for APS1 and rare FGD forms, they may not be available at presentation. Nevertheless, X-linked diseases, including AHC and ALD, and autosomal recessive disorders, including TAS and PHA1b, may present *de novo*, thus not providing physicians a family pedigree at presentation.

Considering disease-specific biochemical markers, these can be identified in APS-1, ALD, and SPL1-related FGD, in which screening for 21-hydroxylase antibodies, VLFA, and sphingolipid intermediates should be performed, respectively. Moreover, it should be reported that sweat test may be useful for differential diagnosis in patients with PHA1, once cystic fibrosis has been excluded.

In patients with diagnosis of rare syndromes associated with AI, surveillance for signs and symptoms of adrenal dysfunction, when not present at diagnosis, should be continued life-long. Accordingly, routine screening for adrenal function should be performed yearly in all patients with APS-1. In patients with cerebral ALD and AMN, yearly testing should be performed until their fourth decade, in which onset of AI becomes rarer [32].

## Management of glucocorticoid and mineralocorticoid replacement

Treatment of acute AI includes fluids and intravenous or intramuscular administration of GC. Adults require hydrocortisone 100 mg followed by 200 mg/day as continuous infusion and tapering to 100 mg/day the following day or until hemodynamic stabilization. In children, along with rapid bolus of intravenous saline, hydrocortisone should be administered with an initial bolus of 50–100 mg/m^2^ followed by 50–100 mg/m^2^/day [85].

Patients with AI require life-long GC and often MC replacement. Current therapy consists of hydrocortisone (HC) and cortisone acetate or prednisone or prednisolone for GC replacement in most patients with AI [85, 87]. In adult patients, suggested dosages are 15–25 mg/daily of HC or HC-equivalent or 3–5 mg/daily of prednisone/prednisolone [87]. The modified-release preparations, Chronocort® (Efmody) and Plenadren® are gaining recognition as they mimic the circadian rhythm with the advantage of only one dose/day (Plenadren) or two doses/day, whereas HC requires three doses/day [88, 89]. In children, the suggested starting treatment dose is HC 8 mg/m^2^ of body surface area. Due to its shorter half-life, HC is preferred over other therapies in children [85], although the immediate-release granule formulation of hydrocortisone, Alkindi® is now licensed for GC replacement in infancy [90–92]. To date, Plenadren is not indicated in patients with CAH [2].

Other specific adjustments may be important for each syndrome. For example, in the context of APS-1, the management of GC replacement requires additional care due to the possible complications related to potential malabsorption due to autoimmune enteritis.

As for the scheme of administration, guidelines suggest splitting total daily GC replacement of immediate-release formulations across the day in two or three doses in adults, with 50% of the daily dosage at awakening and one or two additional doses to be taken until 5 p.m. to mimic the physiological circadian cortisol profile.

In children, three or four administrations are often required to sustain daily activities and growth, leading to impractical fractioning of HC tablets [91]. Recently, newer formulations allowing HC tailoring with palatable granules have been introduced to improve pediatric care of AI [92]. Other proposals that are being developed for pediatric care of AI patients are HC powders [93] and muco-adhesive tablets [94], while modified release and subcutaneous formulations are being tested in children as well [95]. Efmody® has been EMA-approved for children aged > 12 years with congenital adrenal hyperplasia.

All in all, guidelines suggest that children with AI should be treated with the minimum tolerated dose of GC to avoid the detrimental effects of excessive GC exposure on growth, bone and sexual development [85]. In children and adults, tailoring of GC replacement is usually based on clinical signs and symptoms of over- or under-treatment. It is essential that patients and parents are trained in GC replacement principles in the presence of acute illness. The oral stress dose of hydrocortisone for children may be 2–5 × the maintenance dose (depending on the applied sick day protocol) and the intramuscular stress dose depends on the age/body surface of the child: 50 mg/m^2^ or estimate; infants, 25 mg; school-age children, 50 mg; adolescents, 100 mg. Dose adjustments in case of acute illness or concomitant diseases should be performed due to the potential development of life-threatening adrenal crises [85, 96].

Patients with PAI often present or develop MC deficiency, that should be evaluated at baseline by aldosterone and lying renin levels and treated according to electrolyte balance and signs or symptoms such as postural dizziness, salt craving or hypotension [85]. In adults, average doses of fludrocortisone range between 0.05 – 0.2 mg/daily, while in children starting dose is usually 0.05 – 0.1 mg.

In conditions of electrolyte imbalance due to both real MC deficiency or defective MC action in target tissues, hyponatremia, dehydration, and hyperkalemia have to be corrected because of the risk of life-threatening arrhythmias. In the case of severe clinical impairment, the management can require a regimen of intensive care, intravenous salt supplementation with sodium chloride, or sodium bicarbonate if acidosis occurs. When clinical features are mild and the main electrolytic alteration is hyponatremia, oral salt implementation can be sufficient to improve clinical and biochemical aspects. Low potassium diet is generally recommended, but it can be necessary to administrate ionic exchange resins or rarely indomethacin to treat hyperkalemia [70, 71, 73]. In PHA1b, in addition to intravenous correction of electrolytic unbalances, antibiotic treatment and enteral nutrition or gastrostomy in an intensive care setting are required, as systemic complications often occur [70, 73]. On the contrary, as mentioned above, PHA1a is often diagnosed in adults that remained asymptomatic and untreated during their infancy, so that probably the absence of prompt treatment in very mild cases would not really impact survival nor long-term wellbeing [71].

Infants with ADS generally need MC substitution with fludrocortisone and oral sodium supplementation until the normalization of plasma renin activity. Spontaneous improvement or normalization of electrolyte imbalance often occur at 3–4 years of life, and sodium supplementation can be simplified or discontinued during childhood [80, 82, 83].

## Conclusions

Prompt diagnosis and appropriate treatment are essential in newborn and paediatric patients with suspected AI, which remains a life-threatening condition. After exclusion of CAH, a proper clinical and biochemical work-up can lead to suspect rarer forms of AI and select the appropriate gene sequencing. Therefore, a correct and early genetic diagnosis could permit to predict associated clinical features, perform genetic counseling of the families and, in some cases, improve the quality of life of the patients.

## Abbreviations and acronyms

AAD: Autoimmune Addison’s Disease
AHC: Adrenal hypoplasia congenita
AI: Adrenal insufficiency
AIRE: Autoimmune regulator gene
ALD: Adrenoleukodystrophy
AMN: Adrenomyeloneuropathy
APECED: Autoimmune-polyendocrinopathy-candidiasis-ectodermal dystrophy
APS: Autoimmune polyglandular syndromes
ASCT: Allogenic stem cell transplantation
ASD: Aldosterone synthase deficiency
CAH: Congenital adrenal hyperplasia
CARD/HSR: Caspase recruitment domain/homogeneously staining
EnaC: Epithelial sodium channel
FGD: Familial glucocorticoid deficiency
GC: Glucocorticoid
HC: Hydrocortisone
HH: Hypogonadotropic hypogonadism
HypoPTH: Hypoparathyroidism
IUGR: Intrauterine growth restriction
LCAH: Lipoid congenital adrenal hyperplasia
MC: Mineralocorticoid
MC2R: Melanocortin receptor 2
MCM4: Mini-chromosome maintenance complex component 4
MR: Mineralocorticoid receptor
MRAP: Melanocortin receptor 2 accessory protein
NADPH: Nicotinamide adenine dinucleotide phosphate hydrogen
NNT: Nicotinamide nucleotide transhydrogenase
PAI: Primary adrenal insufficiency
PGRMC2: Progesteron Receptor Membrane Component 2
PHA1: Type 1 pseudohypoaldosteronism
PHD: Plant homeodomain
POLE1: Polymerase epsilon‐1
SAI: Secondary adrenal insufficiency
SF-1: Steroidogenic factor 1
S1P: Sphingosine-1-phosphate
SGPL1: Sphingosine-1-phosphate lyase 1
StAR: Steroidogenic acute regulatory protein
TAS: Triple A syndrome
TXNRD2: Thioredoxin reductase 2
VLCFA: Very long chain fatty acids
X-ALD: X-linked adrenoleukodystrophy
